# Moderate and prolonged hypercapnic acidosis may protect against ventilator-induced diaphragmatic dysfunction in healthy piglet: *an in vivo *study

**DOI:** 10.1186/cc12486

**Published:** 2013-01-24

**Authors:** Boris Jung, Mustapha Sebbane, Charlotte Le Goff, Nans Rossel, Gerald Chanques, Emmanuel Futier, Jean-Michel Constantin, Stefan Matecki, Samir Jaber

**Affiliations:** 1 Intensive Care Unit, Department of Anaesthesia and Critical Care, Saint Eloi Teaching Hospital and Institut National de la Santé et de la Recherche Médicale Unit 1046 (INSERM U-1046), Université Montpellier 1, 34295 Montpellier, France; 2 Department of Emergency Medicine, Lapeyronie Teaching Hospital, Université Montpellier 1, 34295 Montpellier, France; 3 Department of Anesthesiology and Critical Care, Clermont Ferrand University Hospital F-63000, France

## Abstract

**Introduction:**

Protective ventilation by using limited airway pressures and ventilation may result in moderate and prolonged hypercapnic acidosis, as often observed in critically ill patients. Because allowing moderate and prolonged hypercapnia may be considered protective measure for the lungs, we hypothesized that moderate and prolonged hypercapnic acidosis may protect the diaphragm against ventilator-induced diaphragmatic dysfunction (VIDD). The aim of our study was to evaluate the effects of moderate and prolonged (72 hours of mechanical ventilation) hypercapnic acidosis on *in vivo *diaphragmatic function.

**Methods:**

Two groups of anesthetized piglets were ventilated during a 72-hour period. Piglets were assigned to the Normocapnia group (*n *= 6), ventilated in normocapnia, or to the Hypercapnia group (*n *= 6), ventilated with moderate hypercapnic acidosis (PaCO_2 _from 55 to 70 mm Hg) during the 72-hour period of the study. Every 12 hours, we measured transdiaphragmatic pressure (Pdi) after bilateral, supramaximal transjugular stimulation of the two phrenic nerves to assess *in vivo *diaphragmatic contractile force. Pressure/frequency curves were drawn after stimulation from 20 to 120 Hz of the phrenic nerves. The protocol was approved by our institutional animal-care committee.

**Results:**

Moderate and prolonged hypercapnic acidosis was well tolerated during the study period. The baseline pressure/frequency curves of the two groups were not significantly different (Pdi at 20 Hz, 32.7 ± 8.7 cm H_2_O, versus 34.4 ± 8.4 cm H_2_O; and at 120 Hz, 56.8 ± 8.7 cm H_2_O versus 60.8 ± 5.7 cm H_2_O, for Normocapnia and Hypercapnia groups, respectively). After 72 hours of ventilation, Pdi decreased by 25% of its baseline value in the Normocapnia group, whereas Pdi did not decrease in the Hypercapnia group.

**Conclusions:**

Moderate and prolonged hypercapnic acidosis limited the occurrence of VIDD during controlled mechanical ventilation in a healthy piglet model. Consequences of moderate and prolonged hypercapnic acidosis should be better explored with further studies before being tested on patients.

## Introduction

Mechanical ventilation is a lifesaving technique and a leading treatment of acute respiratory failure in the intensive care unit (ICU). In the earliest stages of acute respiratory failure, maintaining respiratory muscles at rest, in particular the diaphragm, is frequently performed, the better to synchronize the patient and the ventilator. The ventilator settings often use a totally controlled mode and are combined with deep sedation to avoid spontaneous ventilator cycles. One of the consequences of resting respiratory muscles is the occurrence of ventilator-induced diaphragmatic dysfunction (VIDD) [1-3], largely described in animal models and more sparsely, but recently, in human studies [4-8]. Another complication of mechanical ventilation is the occurrence of ventilator-induced lung injury (VILI), which is magnified when high alveolar pressures and volumes are applied [9], even in healthy lungs [10,11]. As a consequence, the historic goal of mechanical ventilation, normalizing the gas exchange, has evolved toward preventing lung injury by reducing alveolar pressure and lung volume, at the cost of reduced gas exchange. This concept, labeled protective ventilation, may lead to a permissive hypercapnic acidosis, which is well tolerated when not so severe (pH > 7.20 with PaCO_2 _< 60 to 65 mm Hg) [12]. Furthermore, some studies have shown a potential beneficial effect of moderate and prolonged hypercapnic acidosis *per *se on lung inflammation in animal models of experimental pneumonia [13], and it may prevent hypoxia-induced oxydative stress in the lungs [14] and NF-κB activation in pulmonary endothelial cells exposed to lipopolysaccharide [15]. Hypercapnic acidosis may also inhibit endogenous xanthine oxidase activity and then may decrease reactive oxygen species [16]. Conversely, acute and severe hypercapnic acidosis has been shown to impair diaphragmatic contractile properties both *in vitro *[17-19] and *in vivo *[17,20].

Because reactive oxygen species production and the NF-κB pathway play major roles in VIDD [2] and because these pathways can be modulated with hypercapnic acidosis, we conducted an animal study and compared the effects of moderate and prolonged hypercapnic acidosis (72 continuous hours) versus normocapnia in a totally controlled mechanically ventilated healthy piglet model on *in vivo *VIDD. Our hypothesis was that moderate and prolonged hypercapnic acidosis may protect the diaphragm against VIDD.

## Materials and methods

The study followed the guidelines for animal experiments established by the institutional animal care committee (INSERM U 1046, Montpellier, France) and the recommendations of the Helsinki Declaration.

### Animal preparation

We used the same experimental design described in our previous studies [17,21,22]. In brief, 12 piglets (15 to 20 kg) were anesthetized with intravenous pentobarbital sodium (5 to 6 mg/kg). Piglets had their tracheas intubated with a cuffed endotracheal tube, and anesthesia was maintained with continuous intravenous propofol, midazolam, and ketamine without neuromuscular blocking agents. The depth of anesthesia was monitored with bispectral index (BIS; Aspect, Norwood, MA, USA) [23] and was adjusted to block the respiratory drive in both groups. The absence of spontaneous breathing was checked on the ventilator trend graphs, and electromyographic activity of the diaphragm was recorded in a few animals to confirm the absence of electrical activity [17,22].

Volume-controlled ventilation was performed by using an ICU ventilator (Galileo; Hamilton Medical AG, Rhazuns, Switzerland). The following ventilator settings were determined to achieve normocapnia and were maintained throughout the whole experiment: inspired fraction of oxygen (FiO_2_), 0.35; tidal volume (TV), 10 to 12 ml/kg; respiratory rate (RR), 15 to 30 cycles per minute; and positive end-expiratory pressure (PEEP), 5 cm H_2_O. An oral gastric tube and a bladder catheter were placed. Heating pads were used as needed to maintain a normal body temperature of 38.5°C to 39.5°C. Parenteral nutrition was given from the first day, providing 30 to 35 kcal/kg per day. The animals received prophylactic intravenous antibiotics 3 times daily (amoxicillin-clavulanate, 100 mg/kg per day).

After surgical preparation of the right carotid artery, a carotid arterial line (PiCCO; Pulsion, Munich, Germany) was inserted for monitoring of the heart rate, arterial blood pressure, and cardiac output. Arterial and end-tidal CO_2 _partial pressure were checked with a capnograph (Deltatrac; Datex-Ohmeda, Helsinki, Finland) each 4 hours and with arterial blood gases each 12 hours (iSTAT; Abbott, Abbott Park, IL, USA).

A physician provided round-the-clock supervision and animal care for the entire duration of the study. The two groups received the same care, except for the level of capnia.

### Ventilatory care

Animals were separated into two groups in which studied variables were measured each 12 hours: a control group (*n *= 6), mechanically ventilated in normocapnia without any intervention, and a hypercapnia group (*n *= 6), exposed to a determined level of moderate hypercapnic acidosis over a prolonged period. In the hypercapnia group, we increased the instrumental dead space, without any modifications of tidal volume or respiratory rate, to maintain the PaCO_2 _in the range of 55 to 70 mm Hg.

### Assessment of diaphragm muscle activity during mechanical ventilation

#### *In vivo *assessment of transdiaphragmatic pressure

Every 12 hours, we measured transdiaphragmatic pressure (Pdi) to assess *in vivo *diaphragmatic contractile force in both groups, as described in previous studies [17,22]. In brief, double air-filled balloon-tipped catheters were placed transorally into the distal third of the esophagus and in the stomach for measurement of Pdi. Bipolar transvenous pacing catheters were introduced via each internal jugular vein and adjusted to achieve stimulation of the phrenic nerve and subsequent contraction of the diaphragm. Pdi was produced by supramaximal stimulation at frequencies of 20, 40, 60, 80, 100, and 120 Hz in a serial manner. Each train of impulses lasted of 2,000 ms, and each pulse had duration of 150 ms. A pressure-frequency curve was obtained for both groups at each 12-h period and were then compared.

### Statistical analysis

Data are presented as mean ± SD, unless specifically indicated. Normality of the distribution was assessed with the Kolmogorov-Smirnov test.

Comparison of several means was performed by using a repeated-measures analysis of variance and the Newman-Keuls test. A two-way analysis of variance with time (H0, H12, H24,..., to H72) as one factor and modality (normocapnia versus hypercapnia) as the other factor was used. When appropriate, a *post hoc *PLSD Fisher test was used. Nonparametric paired Wilcoxon tests were used to compare data from days 1 and 3 for each animal in both Normocapnia and Hypercapnia groups. All *P *values were two-tailed and a *P *value < 0.05 was considered significant (StatView, version 5.0; SAS Institute Inc., Berkeley, CA, USA).

## Results

### Systemic and biologic response to mechanical ventilation

Long-term mechanical ventilation (that is, 72 hours), either in normocapnia or hypercapnia, did not have consequences on body weight, intestinal transit, diuresis (data not shown) or hemodynamic variables (Table 1). Hypercapnia was associated with an increased cardiac output over time, mainly related to the increase of heart rate. Ventilator parameters were comparable among groups. Surprisingly, PaO_2 _was higher in the Hypercapnia group than in the Normocapnia group, although none of the piglets was hypoxemic. We did not observe any significant differences between the Normocapnia and Hypercapnia groups for all the studied baseline variables. Although BIS values were not significantly different between Normocapnia and Hypercapnia groups (38 ± 8 versus 42 ± 12; *P *> 0.99), mean midazolam-level administration remained at a higher level in the Hypercapnia group (5.1 ± 0.6 mg/h) than in the Normocapnia group (3.5 ± 0.6 mg/h) during the study (*P *< 0.05 between Normocapnia and Hypercapnia groups after 12 hours of ventilation). Doses of propofol were higher in the Hypercapnia group in comparison with the Normocapnia group (102 ± 8 mg/h and 135 ± 49 mg/h in Normocapnia and Hypercapnia groups, respectively, *P *< 0.05 after 12 hours of ventilation).

**Table 1:** Hemodynamic and respiratory variables for the seven steps of measures between the Normocapnia (*n *= 6) and the Hypercapnia (*n *= 6) groups

	H0	H12	H24	H36	H48	H60	H72
Minute ventilation (L/min)							
Normocapnia	5.9 ± 0.8	4.4 ± 0.6	5.1 ± 0.6	4.9 ± 0.5	4.7 ± 0.4	4.8 ± 0.6	4.7 ± 0.8
Hypercapnia	4.6 ± 0.3	4.3 ± 0.1	5.1 ± 0.2	4.8 ± 0.3	4.2 ± 0.2	4.8 ± 0.2	4.3 ± 0.2
Tidal volume (ml)							
Normocapnia	240 ± 40	220 ± 20	230 ± 20	230 ± 25	225 ± 25	230 ± 25	205 ± 10
Hypercapnia	230 ± 22	225 ± 15	235 ± 30	240 ± 15	235 ± 30	230 ± 20	240 ± 20
Respiratory rate (c/min)							
Normocapnia	24 ± 3	20 ± 3	22 ± 2	22 ± 2	21 ± 1	21 ± 1	20 ± 1
Hypercapnia	20 ± 6	19 ± 5	19 ± 4	20 ± 3	18 ± 6	19 ± 4	18 ± 3
Peak pressure (cm H_2_O)							
Normocapnia	22 ± 1	23 ± 2	22 ± 1	21 ± 3	23 ± 2	22 ± 3	23 ± 3
Hypercapnia	22 ± 3	21 ± 2	20 ± 2	22 ± 2	20 ± 3	20 ± 3	20 ± 2
Plateau pressure (cm H_2_O)							
Normocapnia	14 ± 2	14 ± 3	13 ± 2	14 ± 3	13 ± 2	14 ± 4	15 ± 2
Hypercapnia	13 ± 1	14 ± 3	12 ± 3	13 ± 2	12 ± 4	13 ± 3	13 ± 3
pH							
Normocapnia	7.52 ± 0.02	7.47 ± 0.06	7.46 ± 0.04	7.45 ± 0.01	7.44 ± 0.04	7.47 ± 0.04	7.44 ± 0.05
Hypercapnia	7.43 ± 0.07	7.31 ± 0.09^ab^	7.29 ± 0.05^ab^	7.30 ± 0.06^ab^	7.27 ± 0.07^ab^	7.25 ± 0.08^ab^	7.22 ± 0.04^ab^
PaO_2 _(mm Hg)							
Normocapnia	99 ± 8	97 ± 6	95 ± 5	98 ± 3	95 ± 6	97 ± 5	101 ± 6
Hypercapnia	147 ± 8^a^	148 ± 5^a^	149 ± 4^a^	132 ± 4^a^	115 ± 8^a^	146 ± 6^a^	110 ± 9
PaCO_2 _(mm Hg)							
Normocapnia	33 ± 6	39 ± 3	41 ± 3	40 ± 3	39 ± 3	38 ± 2	37 ± 3
Hypercapnia	44 ± 7^a^	65 ± 8^ab^	70 ± 3^ab^	77 ± 8^ab^	73 ± 10^ab^	66 ± 7^ab^	73 ± 4^ab^
SaO_2 _(%)							
Normocapnia	98 ± 2	99 ± 2	97 ± 3	100 ± 2	98 ± 2	97 ± 3	96 ± 4
Hypercapnia	99 ± 2	98 ± 1	97 ± 4	99 ± 2	99 ± 4	98 ± 3	99 ± 3
Plasma bicarbonate (m*M*)							
Normocapnia	27 ± 4	29 ± 4	29 ± 5	29 ± 3	27 ± 3	30 ± 2	26 ± 2
Hypercapnia	28 ± 4	35 ± 5^ab^	33 ± 3^ab^	35 ± 2^ab^	34 ± 5^ab^	32 ± 4^b^	32 ± 5^ab^
Heart rate (c/min)							
Normocapnia	101 ± 10	106 ± 12	107 ± 12	103 ± 10	97 ± 8	101 ± 14	101 ± 15
Hypercapnia	93 ± 18	96 ± 27	91 ± 26	118 ± 26^ab^	123 ± 12^ab^	118 ± 12^ab^	114 ± 16^ab^
Mean blood pressure (mm Hg)							
Normocapnia	75 ± 7	80 ± 10	88 ± 7	86 ± 9	84 ± 13	83 ± 13	88 ± 14
Hypercapnia	83 ± 15	72 ± 14	76 ± 16	87 ± 18	86 ± 22	79 ± 15	77 ± 15
Cardiac Output (l/min)							
Normocapnia	2.2 ± 0.3	2.7 ± 0.3	2.8 ± 0.5	2.8 ± 0.2	2.7 ± 0.2	2.7 ± 0.2	2.7 ± 0.3
Hypercapnia	2.8 ± 0.4	2.9 ± 0.6	3.1 ± 0.6	3.8 ± 0.5^ab^	3.3 ± 0.7^ab^	3.2 ± 0.6^ab^	3.4 ± 0.7^ab^
Stroke volume (ml)							
Normocapnia	22 ± 7	27 ± 10	27 ± 11	28 ± 8	29 ± 10	27 ± 11	26 ± 12
Hypercapnia	28 ± 8	29 ± 12	32 ± 9	28 ± 14	27 ± 8	27 ± 10	30 ± 8

Data are expressed as mean ± SD. ^a^*P *< 0.05 between Normocapnia and Hypercapnia groups; ^b^*P *< 0.05 intragroup difference between time considered and baseline. PaO_2_, arterial pressure of oxygen; PaCO_2_, arterial pressure of carbon dioxide; SaO_2_, oxygen saturation of arterial blood.

### *In vivo *assessment of diaphragmatic force

**Figure 1:**
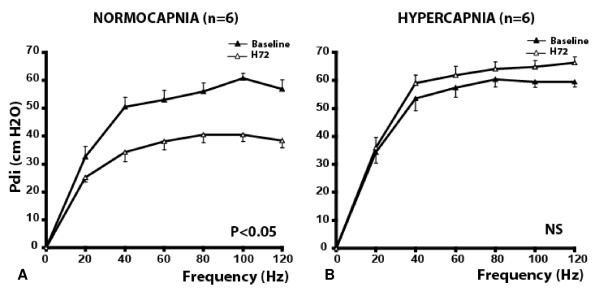
**Diaphragmatic pressure/frequency curves obtained in the Normocapnia group (A) (*n *= 6) and in the Hypercapnia group (B) (*n *= 6)**. Transdiaphragmatic pressure (Pdi) after supramaximal phrenic nerve stimulation at 20, 40, 60, 80, 100, and 120 Hz over a period of 72 hours of mechanical ventilation were obtained in both groups. In the Normocapnia group, Pdi significantly decreased between baseline and H72 at all frequencies (except at 20 Hz) (*P *< 0.05). No significant differences between baseline and H72 were observed for the values obtained at all frequencies in the Hypercapnia group. Data are expressed as mean ± SD. NS, not significant. *P *values refer to between H72 versus baseline.

The baseline pressure-frequency curves of the two groups were not significantly different (Pdi at 20 Hz = 32.7 ± 8.7 cm H_2_O versus 34.4 ± 8.4 cm H_2_O; and at 120 Hz = 56.8 ± 8.7 cm H_2_O versus 60.8 ± 5.7 cm H_2_O, for Normocapnia and Hypercapnia groups, respectively) (Figure 1A and 1B).

**Figure 2:**
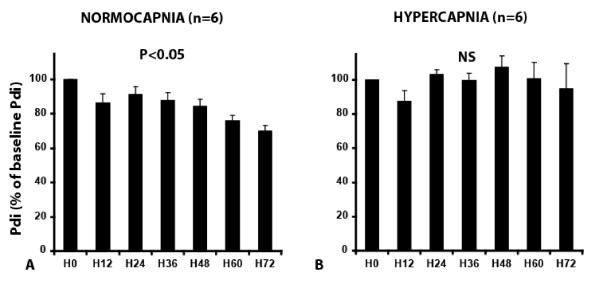
**Transdiaphragmatic pressure (Pdi) expressed as percentage of Pdi at normocapnia obtained at 100 Hz stimulation at each stimulation (every 12 hours) in the Normocapnia group (A) (*n *= 6) and in the Hypercapnia group (B) (*n *= 6)**. Pdi was significantly lower at 100 Hz compared with H0 after 48 hours of mechanical ventilation in the normocapnia group (A). Conversely, no significant decreases were noted in Pdi between the successive measures in the hypercapnia group (B). Data are expressed as percentage (mean ± SD). NS, not significant. *P *values refer to between H72 versus baseline.

Although Pdi decreased significantly in the Normocapnia group between baseline and H72 at all frequencies except 20 Hz (Figure 1A) (*P *< 0.05), it did not change significantly in the Hypercapnia group (Figure 1B). The force decreased significantly after 48 hours of mechanical ventilation in the Normocapnia group, whereas it remained stable in the Hypercapnia group (Figure 2A and 2B).

## Discussion

This study shows that although prolonged totally controlled mechanical ventilation in normocapnia alters diaphragm contractility *in vivo*, maintaining a moderate hypercapnic acidosis during prolonged (72 continuous hours) controlled mechanical ventilation seems to prevent VIDD.

VIDD related to totally controlled mechanical ventilation has been well described, both in several animal studies [17,21,22,24-29] and recently in humans [4-8]. Maintaining diaphragm at rest in the present study (Normocapnia group) effectively promoted VIDD (Figure 1).

Hypercapnic acidosis is frequently observed in critically ill patients. Hypercapnic acidosis can be acute, and as such is often a consequence of an acute disease (acute respiratory failure, hypnotic or opioids overdose), or can be moderate and prolonged, as a consequence of a "protective setting" of mechanical ventilation. The physiologic consequences of hypercapnic acidosis on the brain and the cardiovascular system have been well described and include cerebral hypertension, pulmonary artery vasoconstriction, severe tachycardia, and coma [30,31]. Acute hypercapnic acidosis has been implicated in diaphragm dysfunction in animal studies [17,32-34] as well as in healthy human volunteers [20,35,36]. We recently reported that the recovery of diaphragm contractility impairment related to acute hypercapnic acidosis was incomplete 1 hour after the return to normocapnia [17]. This impairment was associated with the severity of the acidosis and might be partially reversed by inotropes such as dopamine [18] or dobutamine [19]. In opposition to severe and acute hypercapnic acidosis, beneficial effects of moderate hypercapnic acidosis (pH 7.20 to 7.30 and/or PaCO_2 _50 to 70 mm Hg) have been reported on lung inflammation during acute lung injury with or without bacterial pneumonia [13,37-39]. In addition, moderate hypercapnic acidosis may increase cardiac index without increasing oxygen consumption during septic shock [31]. However, few data concern the consequences of moderate hypercapnic acidosis on muscles, in particular, the diaphragm. In the present study, we report for the first time that moderate hypercapnic acidosis seems to prevent VIDD in comparison with normocapnia. Although totally controlled mechanical ventilation with normocapnia during 72 continuous hours led to a 25% decrease of Pdi, evaluated *in vivo *(Figure 1), no significant differences existed between baseline and H72 in the moderate hypercapnic acidosis group. The mechanisms of VIDD related to totally controlled mechanical ventilation have been well described in several animal models, including piglets [22,24,40,41]. The main mechanisms include the stimulation of proteolysis, apoptosis oxidative stress, protein synthesis impairment, autophagy, and sarcomeric lesions [17,28,40-44]. Although those mechanisms have been well explored, very few countermeasures have been reported. Those countermeasures include pharmacologic interventions, such as antioxidative molecules [45-48] or high-dose steroids [49], but also ventilatory measures, such as maintaining spontaneous ventilatory cycles during mechanical ventilation [50-52].

The present study reports that moderate and prolonged hypercapnic acidosis decreases the diaphragm impairment related to prolonged totally controlled mechanical ventilation. The mechanism of the observed "protective" effect of moderate and prolonged acute hypercapnic acidosis is not known and was not explored in the present study.

In the present study, hypercapnic acidosis was associated with an increased cardiac output, even with higher doses of anesthetic drugs (Table 1). Mechanical ventilation *per se *has been reported to impair diaphragm blood flow [53], and whether prolonged and moderate hypercapnic acidosis may prevent this impairment has not been studied. We can speculate that one of the hypothetical mechanisms could be better diaphragm perfusion, although we did not measure the diaphragm blood flow. Another potential mechanism that might have played a role is the increased oxygen delivery to the tissues due to hypercapnic acidosis [54,55]. Moderate hypercapnic acidosis has been demonstrated to possess potent antioxidant properties in the lung, both *in vitro *and *in vivo *[14,16,38], by using biomarkers such as isoprostane or the *in vitro *generation of uric acid from addition of xanthine oxidase to purine in an isolated buffer-perfused rabbit lung [16]. Furthermore, hypocapnic alkalosis significantly increased H_2_O_2_-induced apoptosis and caspase activation of A549 lung cells [56], suggesting that hypocapnic alkalosis may intensify oxidative-induced apoptosis of alveolar epithelial cells via increasing cytosol level of calcium, a strong stimulus of calpain- and caspase-related proteolysis. Regarding those studies, we could speculate that moderate hypercapnic acidosis may inhibit calcium-dependent proteases, thereby limiting the extent of atrophy related to prolonged controlled mechanical ventilation. Moderate hypercapnic acidosis may also promote the transition from glycolytic, type II fibers to type I fibers after several days of hypercapnic acidosis exposure in an animal model [57]. Recently and in opposition to normocapnia, moderate hypercapnia (PaCO_2 _equal to 75 mm Hg) during an 18-hour period of mechanical ventilation in rats was associated with a higher diaphragmatic force, a decrease in diaphragmatic IL-6 and KC pro-inflammatory cytokines, and an increase in diaphragmatic antiinflammatory cytokine IL-10 level [58]. Another well-known effect of moderate hypercapnic acidosis is the inhibition of inflammation process related to mechanical ventilation in healthy or acutely injured lungs [13,38].

Some study limitations must be pointed out. First, we did not compare our results with a control group without anesthesia, or mechanical ventilation, because large animals must be anesthetized for procedures such as phrenic stimulation and Pdi recording.

Second, in the hypercapnia group, to obtain a similar sedation level and to neutralize the centers of breathing, higher levels of propofol and midazolam doses were required. Therefore, we cannot eliminate a direct effect of sedation on Pdi. However, in the clinical situation, sedation is frequently necessary to ensure patient/ventilator synchrony with the controlled mechanical ventilation mode. Although this point clearly represents a methodologic limit of our study, it reflects the clinical interaction between sedation and mechanical ventilation.

Third, PaO_2 _values in the hypercapnia group were slightly above the PaO_2 _levels in the normocapnia group. However, because mean PaO_2 _in the normocapnia group was 350 mm Hg, it is doubtful that this could represent the leading cause of the protective effect observed in the hypercapnia group.

Finally, we do not provide mechanistic analysis for this study.

## Conclusions

The present study reports that prolonged and moderate hypercapnic acidosis may protect against VIDD in a totally controlled mechanically ventilated piglet model.

## Key messages

• Prolonged controlled mechanical ventilation with normocapnia is associated with a diaphragm contractility impairment and ventilator-induced diaphragmatic dysfunction.

• In opposition, prolonged ventilation with moderate hypercapnic acidosis seems to prevent VIDD in a 72-hour healthy piglet study.

## Competing interests

BJ received speaking fees from Merck, not in relation to the present study. EF received speaking fees from GE HealthCare, and from Fresenius Kabi, not in relation to the present study. JMC received speaking fees from LFB, Fresenius Kabi, Merck, Baxter, Draeger, and GE HealthCare, not in relation to the present study. SJ received research grants and speaking fees from Maquet, Draeger, Hamilton Medical, Fisher Paykel, Abbott, not in relation to the present study. Other co-authors have none to declare. Hamilton Medical AG, Rhäzüns, Switzerland supported us, in part, by furnishing the Galileo ventilator.

## Authors' contributions

BJ, SM, and SJ designed the study protocol and wrote the report. CLG, MS, and NR helped in performing the experiments. GC, EF, and JMC helped in correcting the report. All authors read and approved the manuscript for publication.
